# Supplementary material for: Particle image velocimetry of 3D printed vascular fluidic phantom devices
Source: RSC Adv. 2026 Sep 22;16(45):56968–79. doi: 10.1039/d6ra04791b (PMC13596069; doi:10.1039/d6ra04791b)
Supplement: Supplementary file 1 [file RA-016-D6RA04791B-s001.pdf]

Supplementary Information (SI) for RSC Advances.
This journal is © The Royal Society of Chemistry 2026

Supplementary information

Particle Image Velocimetry of 3D printed vascular fluidic phantom devices

Job van Essen, Ahmed Sharaf Denzel Hopman, Selene Pirola and Paola Fanzio

1 Resin parameters

Table 1 Specifications of the Elegoo Mars 5 Ultra MSLA 3D-printer and Anycubic High Clear resin used for the device fabrication

**Resin: Anycubic High Clear**[1]	
**Density**	1.05–1.25 g/cm^3
**Viscosity**	266 cps at 25 °C
**Printer: Elegoo Mars 5 Ultra**[2]	
**X/Y Resolution**	18x18 μm^2
**Layer Height**	10–200 μm
**UV Wavelength**	405 nm
**Light Intensity**	4.0 mW/cm^2
**Build Platform Size**	153.36 × 77.76 × 165 mm^3

2 Rheology of the working fluid

The dynamic viscosity was measured using an Anton Paar MCR302 rotational rheometer with a concentric cylinder (CC39) geometry at angular velocities of 1.5, 3, 6, 12, 24, and 48 RPM. Each measurement was repeated five times. Dynamic viscosity measurements were analyzed using a boxplot (figure 1). In addition, the mean and standard deviation for the dynamic viscosity were calculated at the different RPMs. The boxplot follows the expected trend: the mean dynamic viscosity remains approximately constant up to 12 rpm, after which a noticeable increase occurs. This increase is likely caused by the transition from laminar to secondary flow, where the formation of Taylor vortices introduces measurement errors in the apparent viscosity[3]. At lower rotational speeds, the standard deviation is highest, which is expected since these measurements are close to the lower detection limit of the rheometer, resulting in higher uncertainty[4]. Due to the onset of secondary flow at higher rotational speeds and the increased uncertainty at the lowest speeds, only the measurements in the range of 3–12 rpm were considered for the final analysis. From this range, an average dynamic viscosity of 7.15 mPa·s was obtained.

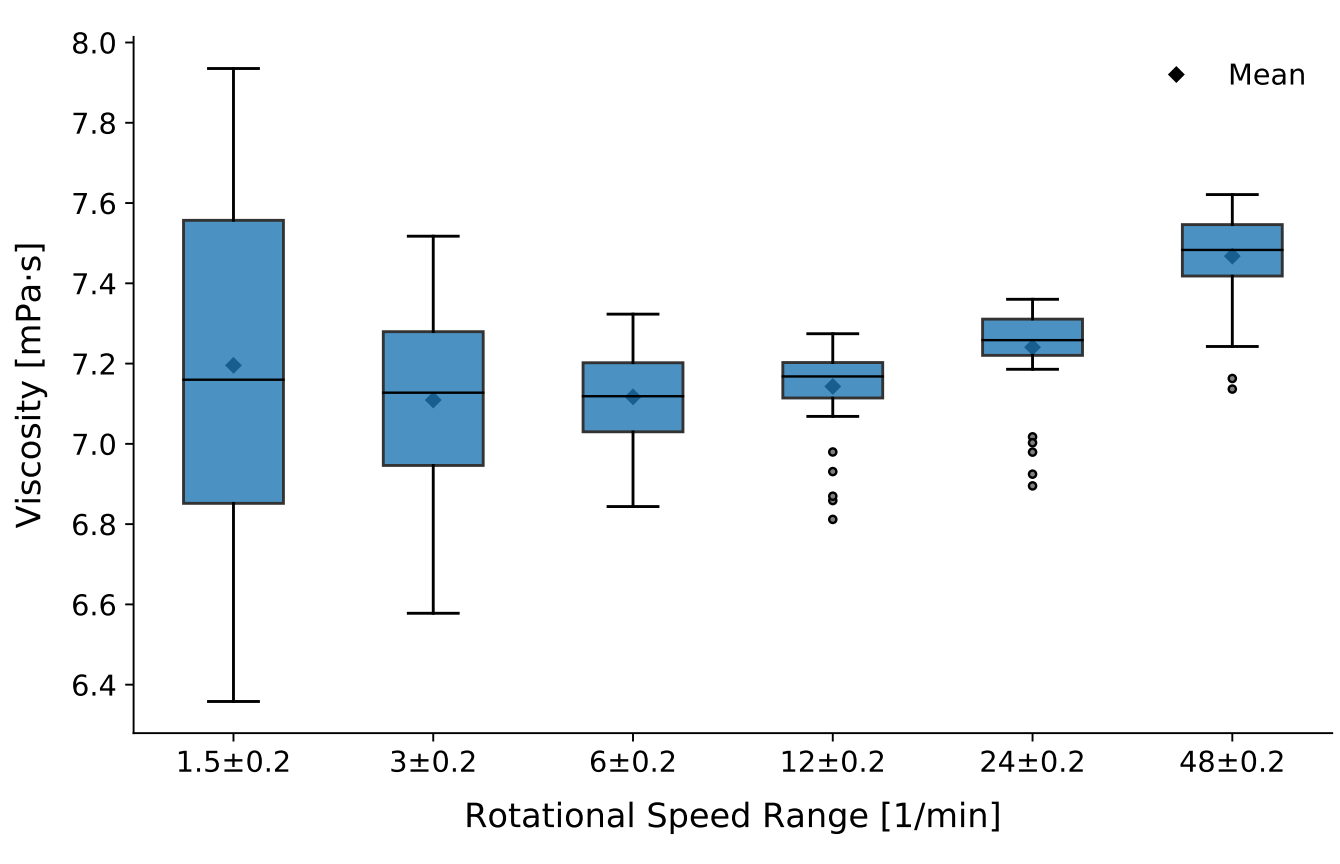

Fig. 1 Boxplot of dynamic viscosity measurements for different rotational speeds. The mean remains consistent between the 3 and 12 rpm, after which the apparent viscosity increases due to the onset of Taylor vortices[3].

3 Channel design

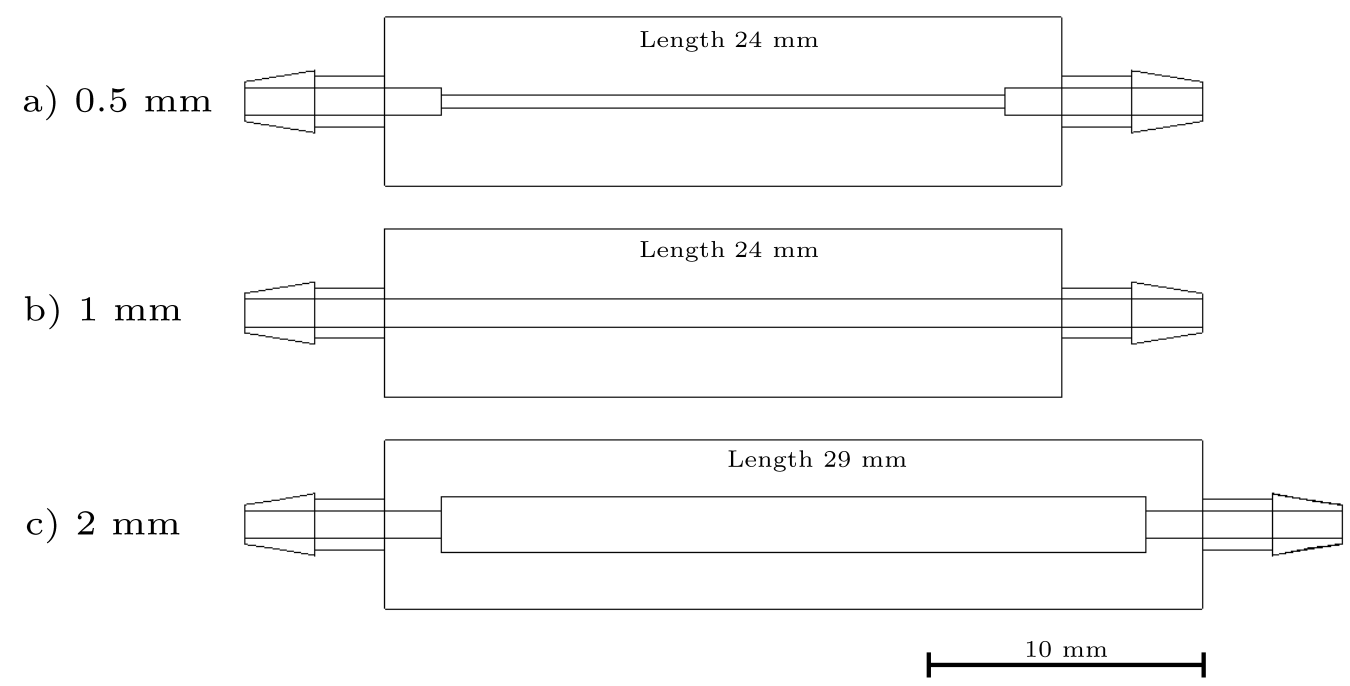

Fig. 2 Computer-aided design (CAD) design of straight channel models with diameters of 0.5 mm, 1.0 mm, and 2.0 mm, each featuring 1 mm inlet and outlet nozzles.

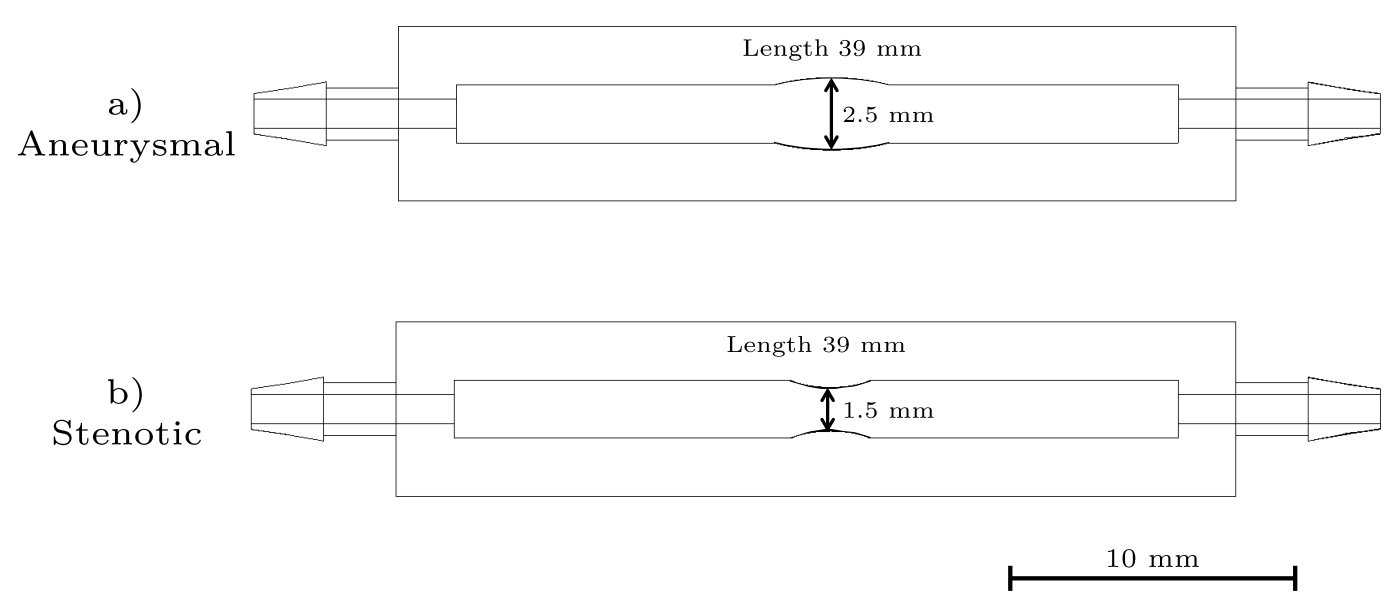

Fig. 3 CAD design of pathological channel models showing (a) an aneurysmal geometry and (b) a stenotic geometry, both scaled 1.25 times relative to a 2 mm reference channel.

4 3D printing parameter optimization

To determine the optimal exposure time for accurate feature replication, the XP2 Validation Matrix has been used. It includes several features such as holes, pins, bars, and grooves. The exposure time has been varied between 1 and 15 seconds. To assess the accuracy of the 3D printed features, the size of the grooves (negative features) and the bars (positive features) has been measured using a digital microscope (Keyence VHX-6000). The measurements were repeated three times to minimize measurement errors and are compared to their intended size as found in the STL file (as measured in SOLIDWORKS).

The graphs, shown in figure 5, contain the exposure time on the X-axis and the measured size on the Y-axis, for the grooves (a)

and the bars (b). The line represents the expected dimension of 1 mm. The results indicate that exposure time plays a crucial role in determining the accuracy of 3D printed features. Shorter exposure times result in incomplete curing or under-exposure, leading to smaller sizes than intended, whereas longer exposure times do the exact opposite. For both the bar and the grooves, an exposure time between 2 and 4 seconds can be used to better match the intended feature size.

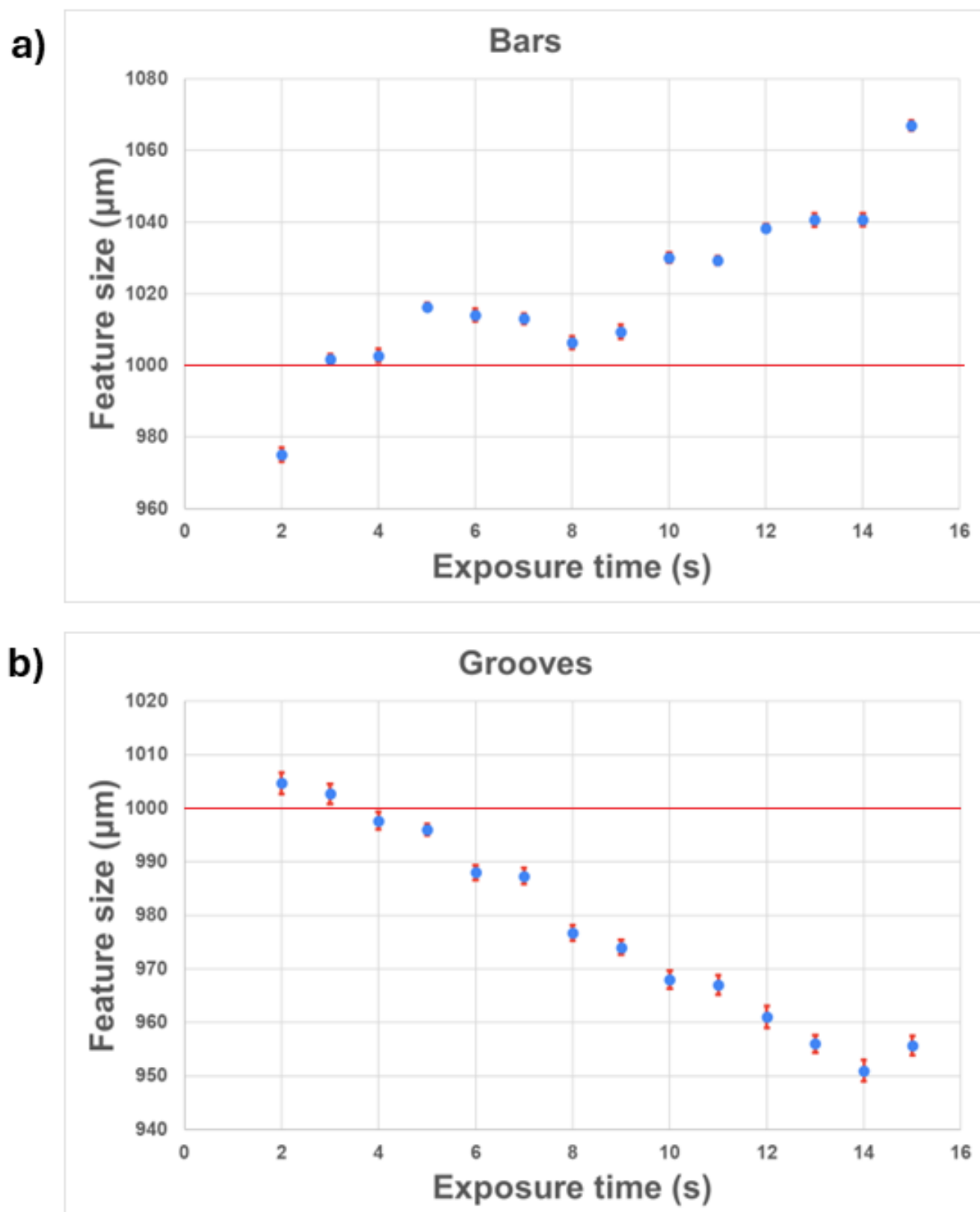

Fig. 4 Feature size versus the exposure time for a) the grooves and b) the bars. The standard error of the mean is calculated for each measurement and added to the graph.

5 Calibration of Flow Sensor

At all five flow rates, the flow sensor calibration procedure is outlined below; each measurement was repeated three times.

1. Weigh the container before starting the calibration
2. Set a fixed flow rate as input in the flow control and wait until steady-state conditions are realized.
3. Capture the output volume for a certain amount of time while monitoring the flow rate sensor output.
4. Weigh the container, which now consists of both the working fluid and the container itself.

The tuning factor could then be determined using Equation 1 and 2.

$$Q_{actual} = \left(\frac{m_{after} - m_{before}}{\rho \cdot t}\right) \quad (1)$$

$$a = \left(\frac{Q_{actual}}{Q_{sensor}}\right) \quad (2)$$

Where Q_{actual} is the actual flow rate, Q_{sensor} is the flow rate output from the sensor, m_{after} is the weight of the sample after the measurements, m_{before} is the weight of the sample before the measurements, ρ is the density of the working fluid, and a is the tuning parameter.

6 Reynolds number calculation

Channe diameter = 0.5 mm	
Flow rate	Re
µl/min	
65	0,6909863
150	1,5945838
240	2,551334
535	5,6873487
950	10,09903

Channe diameter = 1 mm	
Flow rate	Re
µl/min	
65	0,3454931
150	0,7972919
240	1,275667
535	2,8436744
950	5,0495152

Channe diameter = 2mm	
Flow rate	Re
µl/min	
65	0,1727466
150	0,3986459
240	0,6378335
535	1,4218372
950	2,5247576

Fig. 5 Reynolds number for each flow rate/channel diameter combination.

7 Nozzle design

The nozzle design was developed iteratively, balancing printability, connectivity, and leak-proof performance. Figure 6 displays the final CAD design of the nozzle, this design allows for straightforward connection of the microfluidic devices to the flow loop using a silicone tubing (1.4 mm OD and 0.5 mm ID). The nozzle has a 1 mm ID and transitions from 1.4 mm to 2.2 mm OD before narrowing back to 1.8 mm. The total nozzle length is 5.5 mm.

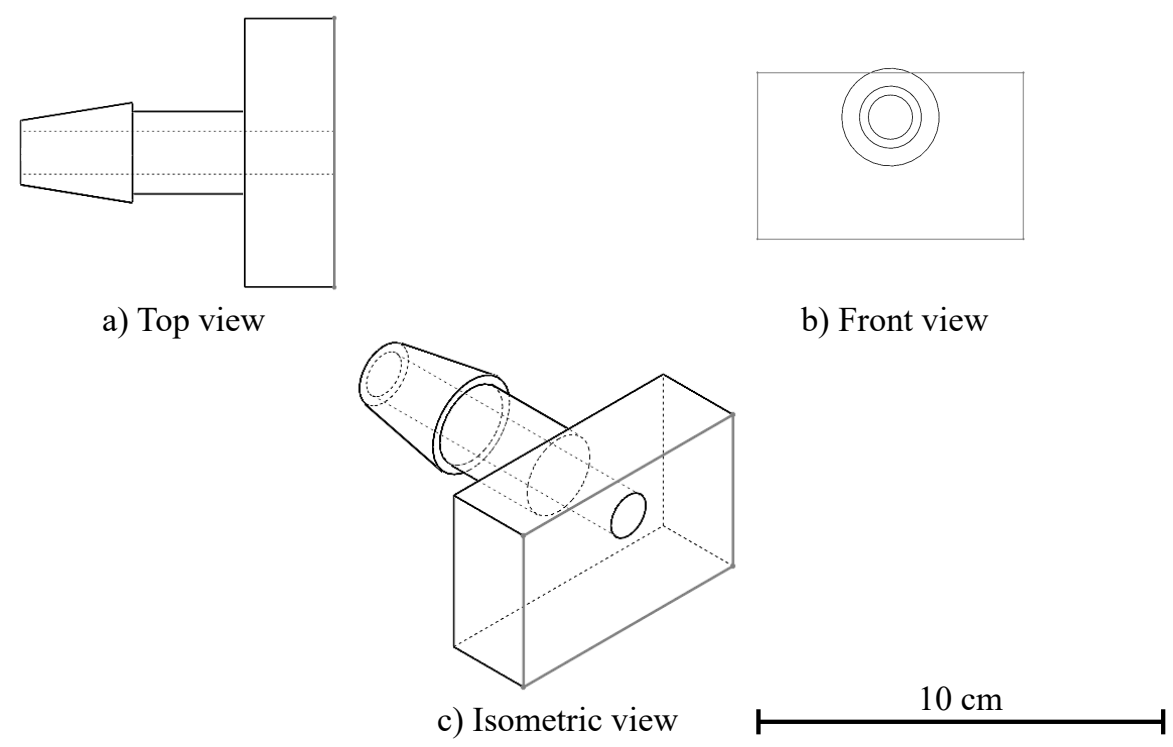

Fig. 6 CAD model of the finalized nozzle geometry showing (a) top, (b) front, and (c) isometric views.

8 Pressure drop measurements

Two Pressure Unit M (–1000 to 1000 mbar range) form Fluigent have been used to measure the pressure drop across the microfluidic device.
The measured pressure difference across the microfluidic device was corrected for tubing losses by subtracting the control measurement (which corresponds to the pressure drop measured for the 100mmFEP tubing).

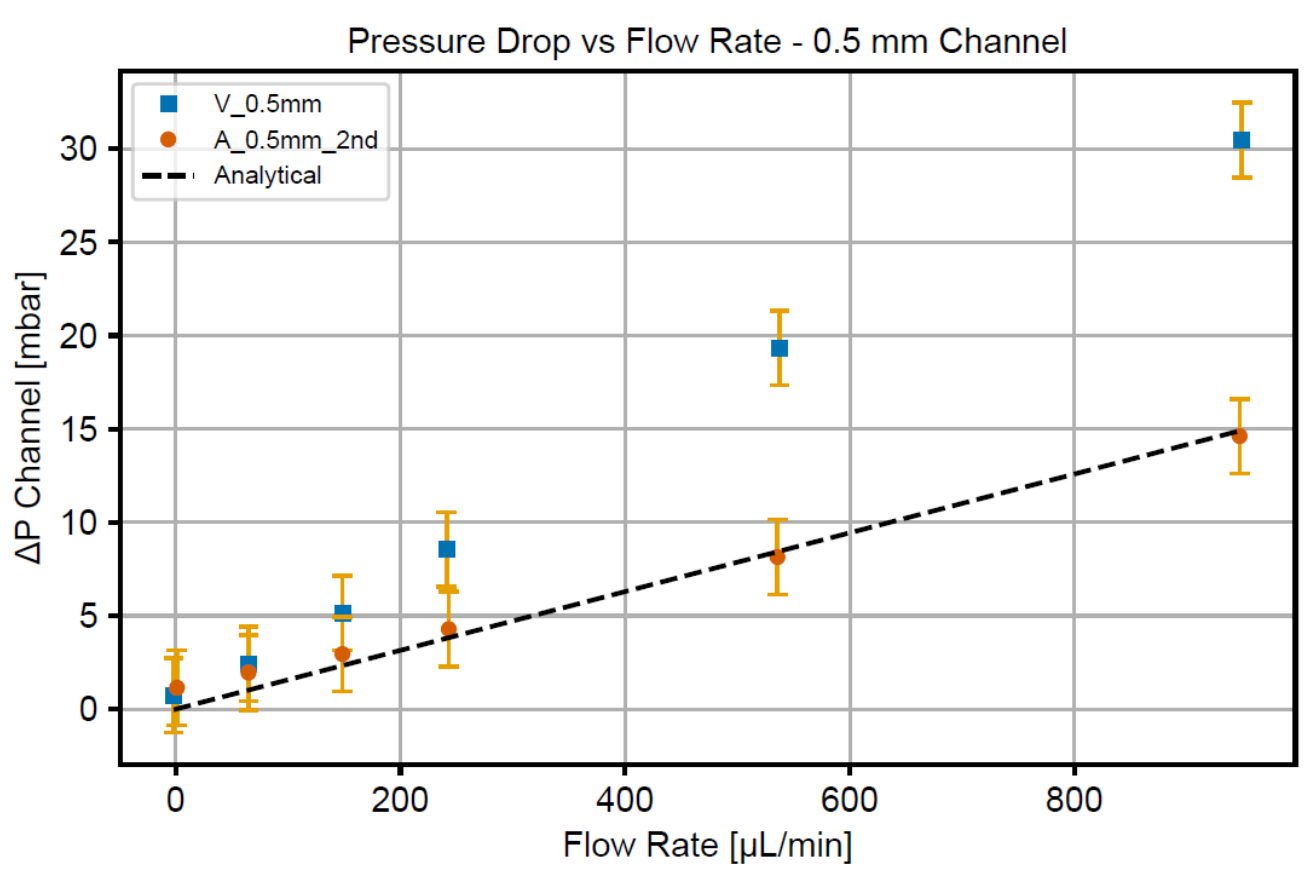

Fig. 7 Measured pressure drop across the angled $500\mu m$ channel and the vertically printed $500\mu m$ compared to the Hagen-Poisseuille solution. Showing that the measurement across the angled $500\mu m$ channel closely follows the Hagen-Poiseuille approximation. The vertically printed channel shows a higher pressure drop compared to the analytical solution (Hagen-Poiseuille). Both channels exhibit a linear relationship between the pressure drop and the flow rate, with a linearity (R2) of 0.996 for the vertically printed channel and 0.980 for the angled printed channel.

Figure 8 shows that the pressure drop for the angled printed 0.5 mm channel closely follows the analytical Hagen–Poiseuille prediction, whereas the vertically printed channel exhibits a higher pressure drop. This deviation can be attributed to the local dimensional errors highlighting the significant impact of channel diameter on hydraulic resistance and, consequently, on the measured pressure drop. The measured pressure drop exhibits a linear relationship with flow rate, with a linearity (R2) of 0.996 for the vertically printed and 0.980 for the angled printed channel. This confirms that the flow remains within the laminar regime, as turbulent flow affects the resistance and consequently the pressure drop. This also indicates that the working fluid behaves as a Newtonian fluid under the conditions investigated. For shear-thinning fluids, a deviation from linearity would be expected, as the apparent viscosity would decrease with increasing flow rate.

9 Wall Shear stress data

Diameter	0.5mm		
	**Angled**	**Vertical**	**Analytical**
**Flow rate (µl/min)**	**WSS [mPa]**	**WSS [mPa]**	**WSS [mPa]**
65	460,43	409,99	623,01
150	1028,79	947,81	1429,17
240	1682,76	1544,83	2343,08
535	3712,79	3412,95	5219,24
950	6580,70	6223,37	9205,71
Average	2693,09	2507,79	3764,04

Diameter	1mm			
	**Angled**	**Analytical**	**Vertical**	**Analytical**
**Flow rate (µl/min)**	**WSS [mPa]**	**WSS [mPa]**	**WSS [mPa]**	**WSS [mPa]**
65	64,93	79,04	69,05	78,34
150	133,18	169,01	159,41	179,60
240	232,10	279,16	272,23	294,67
535			559,53	651,62
950			919,56	1103,83
Average	143,40	175,74	395,96	461,61

Diameter	2mm			
	**Angled 10X**	**Angled 4X**	**Vertical**	**Analytical**
**Flow rate (µl/min)**	**WSS [mPa]**	**WSS [mPa]**	**WSS [mPa]**	**WSS [mPa]**
65	10,10	8,34	12,47	9,80
150	24,99	18,99	29,92	22,47
240	39,91	30,28	48,35	36,81
535	80,35	70,93	104,20	81,46
950	133,45	130,42	186,72	143,60
Average	57,76	51,79	76,33	58,83

Fig. 8 Comparison of all WSS data for each flow rate for straight channels of diameter equal to 0.5mm, 1mm and 2mm

Notes and references

1 Anycubic, *High Clear Resin*, 2025.
2 Elegoo, *Mars 5 Ultra 9K 7-inch Monochrome LCD Resin 3D Printer*, 2025.
3 A. Young and M. Hunt, *Flow transitions and effective properties in multiphase Taylor–Couette flow*, 2021.
4 Anton Paar Wiki, *Avoid measurement errors in rheometry*, n.d.
